# The importance of identifying new and putative target antigens associated with membranous nephropathy: evidence from a Sardinian cohort

**DOI:** 10.1093/ckj/sfaf115

**Published:** 2025-04-18

**Authors:** Nicola Lepori, Andrea Angioi, Benjamin Madden, Matteo Floris, Gianfranca Cabiddu, Doloretta Piras, Paola Bianco, Roberta Mascia, Daniela Onnis, Fernando C Fervenza, Sanjeev Sethi, Antonello Pani

**Affiliations:** Department of Medical Sciences and Public Health, University of Cagliari, Cagliari, Italy; Nephrology, Dialysis and Transplantation, ARNAS Brotzu, Cagliari, Italy; Nephrology, Dialysis and Transplantation, ARNAS Brotzu, Cagliari, Italy; Mayo Clinic Proteomics Core, Mayo Clinic, Rochester, MN, USA; Nephrology, Dialysis and Transplantation, ARNAS Brotzu, Cagliari, Italy; Department of Medical Sciences and Public Health, University of Cagliari, Cagliari, Italy; Nephrology, Dialysis and Transplantation, ARNAS Brotzu, Cagliari, Italy; Nephrology, Dialysis and Transplantation, ARNAS Brotzu, Cagliari, Italy; Pathology Department, ARNAS Brotzu, Cagliari, Italy; Pathology Department, ARNAS Brotzu, Cagliari, Italy; Pathology Department, ARNAS Brotzu, Cagliari, Italy; Division of Nephrology and Hypertension, Mayo Clinic, Rochester, MN, USA; Department of Laboratory Medicine and Pathology, Mayo Clinic, Rochester, MN, USA; Department of Medical Sciences and Public Health, University of Cagliari, Cagliari, Italy; Nephrology, Dialysis and Transplantation, ARNAS Brotzu, Cagliari, Italy

**Keywords:** hyaluronidase 1, mass spectrometry, membranous nephropathy, thrombospondin 1

## Abstract

**Background:**

Membranous nephropathy (MN) is a leading cause of nephrotic syndrome (NS). Since the identification of anti–phospholipase A2 receptor (anti-PLA2R) antibodies in 2009, the use of laser microdissection and tandem mass spectrometry (LMD/MS) has allowed the discovery of several target antigens in MN.

**Methods:**

In this retrospective cohort study, adult patients evaluated at the Division of Nephrology at Brotzu Hospital (Cagliari, Italy) with biopsy-proven MN and a negative serological test for anti-PLA2R antibody underwent LMD/MS, performed at the Department of Laboratory Medicine and Pathology of Mayo Clinic (Rochester, MN, USA).

**Results:**

Twenty-four cases of biopsy-proven MN were available for antigen detection by LMD/MS studies. High total spectral counts of PLA2R were detected in 12 out of 24 (50%) cases. In addition, high spectral counts of THSD7A and NELL1 were detected in two cases each, and EXT1/EXT2 and NCAM1 in one case each. Five putative antigens have been detected: SULF1, PGLYRP, HYAL1, THBS and SEZ6L2.

**Conclusions:**

Our study highlights at least two interesting considerations. First, the determination of PLA2R on renal tissue in the diagnosis of PLA2R-associated MN is emphasized since 50% of our cases were falsely diagnosed with PLA2R-negative MN based on the serum anti-PLA2R antibodies determination. Second, our study shows six patients with MN likely associated with putative antigens, two of them showing new antigens never described before in literature (HYAL1 and THBS1). This high prevalence of putative antigens in our cohort is not easily explainable and paves the way for evaluating specific factors in the Sardinian population that could explain this evidence.

## Graphical Abstract

**Graphical Abstract:**
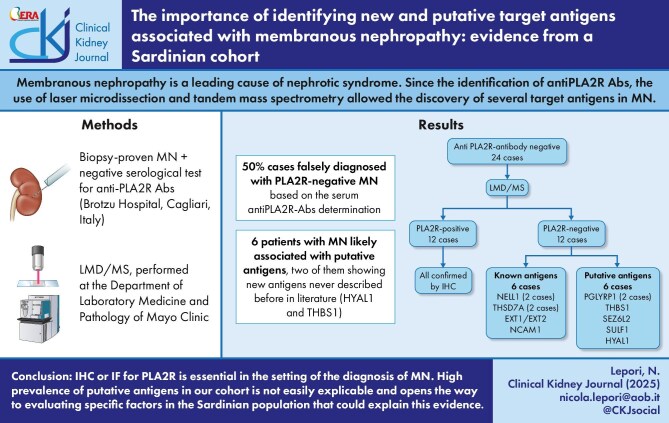

KEY LEARNING POINTS **What was known:**

- The discovery of new target antigens in membranous nephropathy (MN) has challenged the traditional classification of MN into primary and secondary forms; at least 14 target antigens have been identified, accounting for 80%–90% of cases of MN.
- The term “putative antigens” describes proteins identified on laser microdissection and tandem mass spectrometry (LMD/MS) in cases of MN negative for several already known antigens.
- Although 10% of MN cases could be associated with “putative antigens,” each of them, when considered individually, is extremely rare (<2%).

**This study adds:**

- This study shows a high prevalence of putative antigens in a small Sardinian cohort of MN patients.
- Two new potential putative antigens are described for the first time (HYAL1 and THBS1)

**Potential impact:**

- Further efforts to widen the diffusion of LMD/MS could lead to a better definition of MN target antigens.

## INTRODUCTION

Membranous nephropathy (MN) is a leading cause of nephrotic syndrome (NS) in White adults and the second cause of NS in African American and Hispanic patients. MN carries a high disease burden, with up to 16%–40% of patients developing

kidney failure at 5–15 years [1]. The diagnosis of MN is based on the finding of bright granular immunoglobulin G (IgG) along the glomerular basement membrane on immunofluorescence microscopy (IF) and subepithelial electron-dense deposits on electron microscopy. Traditionally, MN has been classified as “primary” when there is no known associated disease, or “secondary” when there is disease association with autoimmune disease, malignancy, drugs, infection, hematopoietic stem cell transplant and demyelinating polyneuropathy. In 2009, a podocyte protein, phospholipase A2 receptor (PLA2R), was identified as causal antigen of primary MN, showing autoantibodies against PLA2R (anti-PLA2R-Abs) in 70% of cases [2]. Subsequently, in 2014, a second antigen, thrombospondin type 1 domain-containing 7A (THSD7A), was identified in 1%–5% of MN cases [3]. Recently, using laser microdissection and tandem mass spectrometry (LMD/MS), several target antigens have been described in MN, including exostosin 1/2 (EXT1/2), neural epidermal growth factor-like 1 (NELL1), serine protease HTRA1 (HTRA1), semaphorin 3B (SEMA-3B), protocadherin 7A (PCDH7A), protocadherin FAT1 (FAT1), netrin G1 (NTNG1), contactin 1 (CNTN1), neural cell adhesion molecule-1 (NCAM1), transforming growth factor beta receptor 3 (TGFBR3), proprotein convertase subtilisin/kexin type 6 (PCSK6) and neuron-derived neurotrophic factor (NDNF) [4–13]. The discovery of these antigens associated with MN challenges the historical classification (primary vs secondary) because a target antigen can now be identified in approximately 80% of MN [14, 15].

We performed a retrospective study on a cohort of MN patients with negative anti-PLA2R-Abs test, using mass spectrometry in order to determine the specific antigen in these cases. We analyzed the clinical and serological features of the three different groups obtained: PLA2R-associated MN, PLA2R-negative MN in which an antigen was detected, and PLA2R-negative MN in which a “putative” antigen was detected.

## MATERIALS AND METHODS

In this retrospective cohort study, all adult patients evaluated at the Division of Nephrology at Brotzu Hospital (Cagliari, Italy) with biopsy-proven MN and a negative serological test for anti-PLA2R-Ab were identified. Laser microdissection and tandem mass spectrometry (LMD/MS) were performed at the Department of Laboratory Medicine and Pathology of Mayo Clinic (Rochester, MN, USA) to determine the MN antigens in the PLA2R-negative MN cohort. Clinical and biopsy findings were obtained from the charts.

### Clinical data

Demographic characteristics, kidney function, proteinuria and medication history were recorded at the time of kidney biopsy. Medical records were obtained for malignancies, autoimmune diseases, infectious diseases and drug use. Laboratory data were reviewed for antinuclear Abs (ANAs), anti-double-stranded DNA (anti-dsDNA) Abs, other autoimmune serology, complement factors, hepatitis serology and paraproteinemia evaluation. Follow-up data on the evolution and treatment of the MN and any identified associated disease were collected. Remission of MN was defined as complete if proteinuria was 0.3 g/24 h or less, partial if proteinuria was >0.3 but <3.5 g/24 h, and no remission if proteinuria was 3.5 g/24 h or greater within 1 year of MN diagnosis. In the absence of a 24-h urine collection, a random urine protein–creatinine ratio was used. Remission was categorized as spontaneous, induced by immunosuppression, induced by treatment of the associated condition, or withdrawal of the offending drug.

### Anti-PLA2R-Abs tests

Anti-PLA2R-Abs were tested using immunofluorescence assay (IFA) until 2015; from 2016 onwards enzyme-linked immunosorbent assay (ELISA) was used (EUROIMMUN, Lübeck, Germany). IFA was considered negative when the Ab titer was less than 1:10. ELISA was considered negative, as per manufacturer's instructions, when Ab concentration was <14 RU/mL.

### Renal biopsies processing

Standard renal biopsy processing techniques were used, including light microscopy, immunofluorescence and immunohistochemistry. All light microscopy samples were stained with hematoxylin and eosin, Jones methenamine silver, Masson trichrome and periodic acid–Schiff reagent. Direct immunofluorescence was performed on neutral buffered formalin-fixed, paraffin-embedded tissue. Sections were cut at 3 μm and deparaffinized, and after blocking endogenous peroxidase were incubated with Proteinase K (Leica) and reacted with fluorescein-tagged polyclonal rabbit anti-human Abs to IgG, IgA, IgM, C3, C4, C1q, fibrinogen, and *κ*- and *λ*-light chains (Agilent, Dako) for 30 min; a cover slip was applied using aqueous mounting media. Biopsies samples were stained with immunohistochemistry Ab for IgG4 (clone MRQ-44 Mouse monoclonal Ab, Cell Marque, Sigma-Aldrich), C4d (rabbit anti-human polyclonal Ab RTU, Vitro Master Diagnostica) and anti-PLA2R1 (rabbit anti-human polyclonal Ab, Sigma-Aldrich 1:200). All biopsy samples were stained [immunohistochemistry (IHC) and fluorescin isothiocyanate (FITC)] using a fully automated staining system (Leica BOND III).

### Laser microdissection and tandem mass spectrometry

LMD/MS has been described previously [4, 16]. Briefly, for each case, 10-µm thick formalin-fixed paraffin sections (FFPE) were obtained and mounted on a special PEN membrane laser microdissection slide, and using a Zeiss Palm Microbean microscope, the glomeruli were microdissected to reach approximately 250–550 000 μM^2^ per case. The resulting FFPE fragments were digested with trypsin and collected for MS/MS analysis. The trypsin-digested peptides were identified by nano-flow liquid chromatography electrospray tandem MS/MS (nanoLC-ESI-MS/MS) using a Thermo Scientific Q-Exactive or Exploris480 Mass Spectrometer (Thermo Fisher Scientific, Bremen, Germany) coupled to a Thermo Ultimate 3000 RSLCnano HPLC system. All MS/MS samples were analyzed using Mascot and X! Tandem set up to search a SwissProt human database. Scaffold (version 4.8.3, Proteome Software Inc., Portland, OR, USA) was used to validate MS/MS-based peptide and protein identifications. Peptide identifications were accepted at >95.0% probability by the Scaffold Local FDR algorithm with protein identifications requiring a 2-peptide minimum and a 95% probability using Protein Prophet.

## RESULTS

Thirty-one cases of biopsy-proven MN in patients with no detectable circulating anti-PLA2R-Ab diagnosed at our Renal Unit from 2008 to 2021 were identified. Kidney biopsy tissue was available for antigen detection by LMD/MS studies in 24 cases. The test for anti-PLA2R-Abs was performed in five cases with the use of IFA and in 19 cases with the use of ELISA. Among patients studied with IFA, three cases were analyzed at the onset of disease, while two cases were analyzed during a relapse. Among patients studied with ELISA, 13 cases were analyzed at the onset of the disease, while 6 cases were analyzed during relapse. The clinical and histological features of the cohort are reported in Table 1.

**Table 1:** Characteristics of 24 cases of serum-negative anti-PLA2R MN.

	Serum anti-PLA2R-negative MN (*N* 24)
Age (years), mean ± SD	47.5 ± 20.9
eGFR (mL/min), mean ± SD	91.3 ± 41.7
Proteinuria (g/24 h), mean ± SD	6.95 ± 3.0
Albuminemia (g/dL), mean ± SD	2.7 ± 1.0
Nephrotic syndrome, % (*n*)	83.3% (20)
History of autoimmune disease, % (*n*)	29% (7)
ANA and/or ENA and/or anti-dsDNA positive, % (*n*)	29% (7)
History of malignancy, % (*n*)	16.6% (4)
Active malignancy, % (*n*)	8.3% (2)
HBsAg positive, % (*n*)	4.1% (1)
Anti-HBc positive, HBsAg negative, % (n)	33.3% (8)
Anti-HCV positive, % (*n*)	0% (0)
Relapsing patients, % (*n*)	37.5% (9)
Spontaneous remission, % (*n*)	29.1% (7)
IS treatment, % (*n*)	87.5% (22)
Complete + partial remission, % (*n*)	95.8% (23)
IgG + C3 on IF, % (*n*)	54.1% (13)
Full house on IF, % (*n*)	16.6% (4)
Only IgG on IF, % (*n*)	12.5% (3)

eGFR, estimated glomerular filtration rate; SD, standard deviation; IS, immunosuppression.

### Laser microdissection and mass spectrometry (LMD/MS)

The following MN antigens were detected by LMD/MS (Table 2).

(i) Known antigens: high total spectral counts of PLA2R were detected in 12 of the 24 (50%) cases. The mean spectral count was 59.3 (± 20.9). In addition, high spectral counts of THSD7A and NELL1 were detected in two cases each, and EXT1/EXT2 and NCAM1 in one case each.
(ii) Putative antigens: high total spectral counts of sulfatase 1 (SULF1; one case), peptidoglycan recognition protein 1 (PGLYRP1; two cases), hyaluronidase 1 (HYAL1; one case), thrombospondin 1 (THBS1) (one case) and seizure related 6 homolog like 2 (SEZ6L2; one case) were detected.

**Table 2:** LMD/MS results in 24 PLA2R-Ab negative MN.

Patient	Number of glomeruli	Square area	Antigen	Total spectral count	Log2 iBAQ value
1	53	766 652	PGLYRP1	2	21.09
2	33	524 205	EXT1/EXT2	33/41	24.90/25.52
3	29	768 351	SEZ6L2	25	25.91
4	32	774 117	NCAM1	42	26.64
5	6	134 603	HYAL1	27	24.43
6	22	371 559	THSD7A	86	25.74
7	47	756 075	THSD7A	41	24.52
8	34	597 548	THBS1	51	25.29
9	7	113 580	NELL1	40	24.36
10	27	491 912	PGLYRP1	2	20.2
11	40	763 397	NELL1	33	25.37
12	23	352 075	SULF1	24	23.66
13	27	325 240	PLA2R	51	23.60
14	21	369 304	PLA2R	84	25.41
15	20	264 207	PLA2R	64	25.07
16	6	121 143	PLA2R	41	23.02
17	37	744 977	PLA2R	54	24.71
18	21	694 000	PLA2R	53	24.64
19	9	195 926	PLA2R	45	23.46
20	31	751 016	PLA2R	97	26.63
21	27	767 355	PLA2R	69	25.61
22	47	694 632	PLA2R	17	23.59
23	28	617 062	PLA2R	66	25.47
24	27	681 254	PLA2R	71	25.76

### Immunohistochemistry

**Figure 1:**
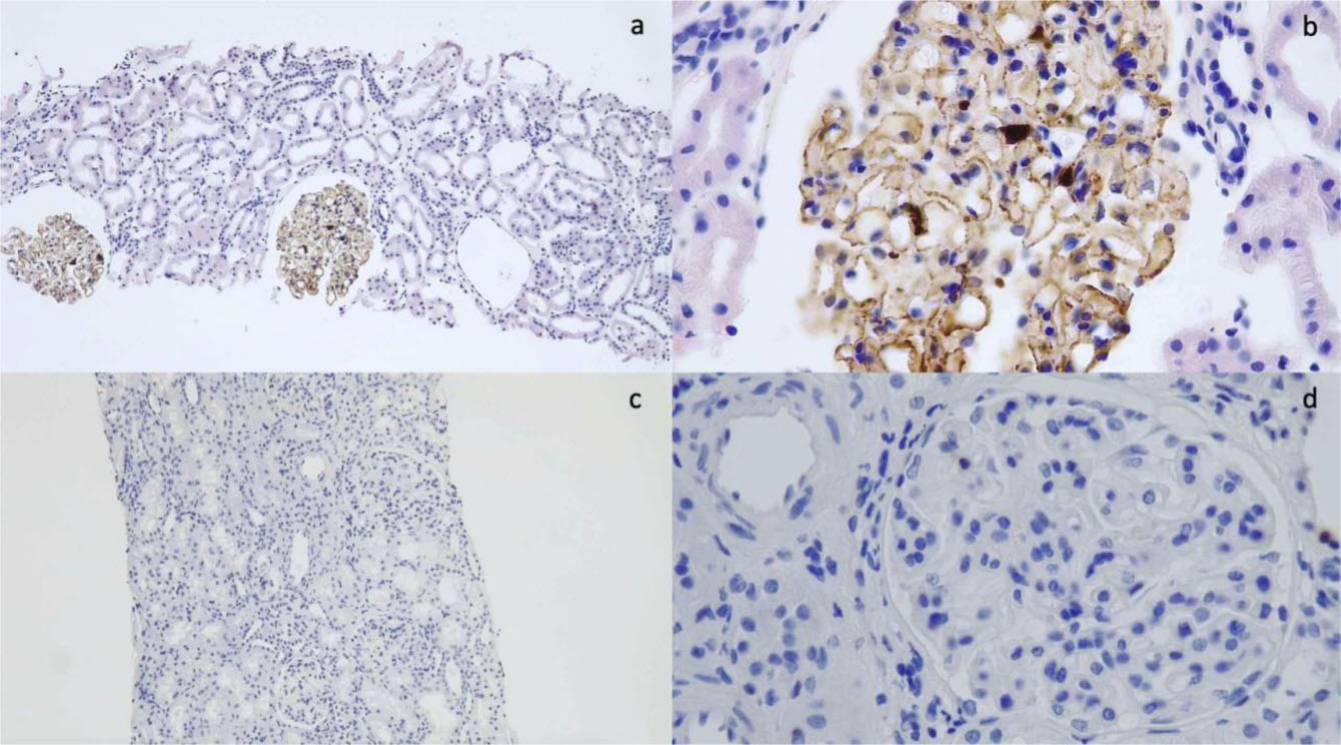
(**a**) PLA2R immunohistochemistry 10×, global and diffuse granular positivity along glomerular basement membrane. (**b**) PLA2R immunohistochemistry 40×, global and diffuse granular positivity along glomerular basement membrane. (**c**) PLA2R immunohistochemistry 10×; negative staining. (**d**) PL2AR immunohistochemistry 40×; negative staining.

IHC was performed for PLA2R in the 24 cases with no detectable circulating anti-PLA2R-Ab; 12 of them showed granular positivity along the glomerular basement membrane for PLA2R (Fig. 1a and b), confirming results from LMD/MS, and the other 12 were completely negative (Fig. 1c and d). For each stain, a score was given on a scale of 0–3+, along with a pattern of positivity, focal or diffuse, and segmental or global. Cases of minimal change disease were taken as negative control.

### Clinical findings and outcomes

**Figure 2:**
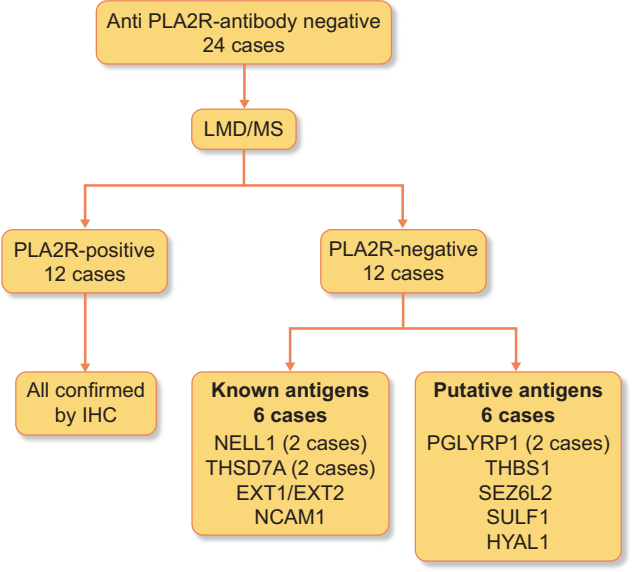
Classification of MN cases in our cohort according LMD/MS results.

Based on the LMD/MS and IHC results, our cohort was reclassified into two groups: 12 patients were diagnosed with PLA2R-associated MN and 12 patients were diagnosed with PLA2R-negative MN (Fig. 2). Clinical features, histological features and responses to treatment of the two subgroups are reported in Table 3. Among those patients who were positive for PLA2R on IHC, 2 were tested for anti-PLA2R-Abs with immunofluorescence (1 at the onset of disease and 1 during a relapse), while 10 were tested for anti-PLA2R-Abs with ELISA (6 at the onset and 4 during a relapse): in the latter subgroup, 6/10 patients showed anti-PLA2R concentration <14 RU/mL but >2 RU/mL. Among the 12 patients with negative PLA2R IHC and negative PLA2R on LMD/MS, 6 patients showed known MN antigens on MS/MS, and 6 patients showed new putative antigens on MN. The cases were divided based on disease-associated, clinical and pathologic findings, and antigen detected (complete data available in Supplementary data, Table S1).

(i) Five patients showed features of autoimmune disease (known autoimmune diseases and/or positive serology). Two female patients (age 29–52 years) had a diagnosis of systemic autoimmune disease (one case of systemic lupus erythematosus and one case of rheumatoid arthritis): both showed a full-house pattern on IF (IgG, IgA, IgM, C3 and C1q positive). ANA were positive in both patients, who showed anti-dsDNA and extractable nuclear antigen (ENA)-SSA positivity, respectively. Two male patients, with no known history of autoimmune diseases, showed evidence of rich IF on renal biopsy (IgG, IgA, IgM and complement) and positive circulating autoantibodies (both patients had positive ANA and anti-dsDNA, one showed low serum C3 and C4): no clinical evidence of systemic autoimmune disease were found at time of diagnosis. One patient with a medical history of alopecia, autoimmune thrombocytopenia, and autoimmune hemolytic anemia had MN diagnosis at the age of 16 years. In this subgroup, one patient was EXT1/EXT2 positive, and one patient was NCAM1 positive. The three remaining patients showed moderate to high spectral counts on LMD/MS for proteins that could be considered putative antigens for MN: PGLYRP1 and SEZ6L2, which have been already described as putative antigens in MN, and HYAL1, is a protein that is not yet described in literature for its association with MN.
(ii) Two patients showed a history of malignancy. One female patient had a history of renal cell carcinoma treated with tumor resection 18 months before MN onset, and a male patient had a diagnosis of focal adenocarcinoma of the large bowel during the screening performed after MN diagnosis. Both patients showed LMD/MS positive for THSD7A.
(iii) One patient developed NS at the age of 22 years, during the 25th week of a first pregnancy. She was initially treated with steroids, without achieving a complete remission; therefore, 7 weeks after the delivery, a renal biopsy was performed: LMD/MS showed moderate to high spectral count for THBS1, a putative antigen previously not described in literature.
(iv) The remaining four patients (two males, two females) were aged between 57 and 81 years: history of autoimmune disease and history of malignancies were both negative. Two patients showed LMD/MS positive for NELL1, and two patients showed LMD/MS positivity for putative antigens (both previously described in literature: PGLYRP1 and SULF1).

**Table 3:** Characteristics of PLA2R-associated MN vs PLA2R-negative in our cohort.

	PLA2R associated (*N* 12)	PLA2R negative (*N* 12)	*P*-value
Age (years), mean ± SD	42.9 ± 19.4	52.16 ± 23	.28
Male sex (%)	75%	50%	.19
eGFR (mL/min), mean ± SD	96.3 ± 41.7	86.25 ± 45	.67
Proteinuria (g/24 h), mean ± SD	6.75 ± 3.0	7.16 ± 3.2	.59
Albuminemia (g/dL), mean ± SD	2.9 ± 0.59	2.56 ± 0.66	.53
Nephrotic syndrome (%)	83%	83%	.48
History of autoimmune disease (%)	25%	33.3%	.69
ANA and/or ENA and/or anti-dsDNA positive (%)	**8.3%**	**41.6%**	**.05**
History of malignancy (%)	16.6%	16.6%	1
Active malignancy (%)	0%	7.3%	.39
HBsAg positive (%)	8.3%	0%	.25
antiHBc positive, HBsAg negative (%)	33.3%	33.3%	1
Anti HCV positive (%)	0%	0%	1
**Relapsing patients (%)**	**58.3%**	**16.6%**	**.03**
Spontaneous remission (%)	25%	33.3%	.87
IS treatment (%)	83.3%	91.6%	.78
Complete + partial remission	100	94.1	.89
IgG + C3 on IF (%)	75%	33.3%	.27
Full house on IF (%)	0%	33.3%	.35
Only IgG on IF (%)	0%	25%	.43

eGFR, estimated glomerular filtration rate; SD, standard deviation; IS, immunosuppression.

Bold has been used when difference among groups is statistically significative.

## DISCUSSION

In recent years, up to now, glomerular microdissection and mass spectrophotometry have led to the identification of numerous antigens associated with MN [17]. The discovery of PLA2R and many other antigens associated with MN allowed a group of expert pathologists and clinical nephrologists, who met in October 2022 at the Mayo Clinic in Rochester (MN, USA) to produce and recently publish [14, 15] a consensus document proposing a new classification of MN, fundamentally based on two phases. The first is the association of MN with a specific antigen, and the second is the association with the concurrent clinical conditions.

Considering that in our center, as in most Italian and European centers involved in the routine clinical diagnosis of glomerulopathies, mass spectrophotometry techniques are not routinely performed, we retrospectively re-analyzed a cohort of 31 MN patients considered PLA2R negative (due to the absence of serum anti-PLA2R-Abs), performing glomerular microdissection and mass spectrophotometry.

The first interesting result is the high number of patients who showed LMD/MS positivity for PLA2R despite the absence of circulating anti-PLA2R-Abs (12/24, 50%). These data are consistent with what has been previously reported in the literature: Debiec and Ronco in 2011 described that 24% of patients who did not have circulating Abs had the PLA2R antigen detected within immune deposits by immunofluorescence of the biopsy specimen [18]. Several subsequent studies have confirmed that the tissue determination of PLA2R is a more sensitive tool, compared with the search for circulating Abs, in the diagnosis of PLA2R-associated membranous nephropathy [19, 20]. Two possible explanations have been commonly proposed. Anti-PLA2R-Abs, if tested in a phase of immunological remission that precedes clinical remission, could result falsely negative as clearly demonstrated by Svobodova *et al*. in 2013 [21]. The second possibility is that in an early phase of the disease, circulating Abs are not yet identifiable in the patient's serum because they are absorbed by the glomerular immune deposits (the so-called “kidney as sink” hypothesis). This hypothesis is supported by data from two small case series published by van de Logt [22] and Ramachandran [23]. It is also necessary to underline the role of IFA in those patients with ELISA showing anti-PLA2R-Ab values <20 RU/mL and >2 RU/mL. In the Mayo Clinic experience published in 2021 all patients with ELISA values >2 RU/mL and <20 RU/mL and a positive IFA were found to have MN on the biopsy, while only 57.5% of the patients with ELISA values between >2 and <20 RU/mL and a negative IFA had MN confirmed by the biopsy [24]. Our results therefore highlight the utmost importance of the integration of ELISA results with IFA and research for PLA2R on renal tissue in order to avoid missing a non-negligible percentage of PLA2R-associated MN.

Six patients with PLA2R-negative MN (serum and IHC negative) showed moderate to high spectral counts on LMD/MS positive for proteins already well known as antigens in MN: two THSD7A-associated MN, two NELL1-associated MN cases, one NCAM1-associated MN and one EXT1/EXT2-associated MN have been diagnosed. The most significant data regarding this subgroup emerge from the analysis of the clinical and histological characteristics observed in these patients. The two cases of THSD7A-associated MN showed, in fact, a strong correlation with neoplastic pathology: one case of adenocarcinoma and one case of renal neoplasia. As reported in the literature, a substantial minority of patients with THSD7A-associated MN have a concurrent malignancy; a large series showed malignancy in 31 of 195 patients (16%) with THSD7A-associated MN [25]. The only EXT1/EXT2-associated MN case and the only NCAM1-associated MN diagnosed in our cohort were observed in patients who showed positive circulating Abs (anti-dsDNA, ANA) associated with a full-house pattern on IF. These data are strongly consistent with the evidence available in the literature, which shows that EXT1/EXT2 and NCAM1 are the two antigens most frequently observed in lupus membranous nephritis and MN associated with autoimmune diseases [26, 27]. The two cases of NELL1-associated MN observed in our cohort do not show any associated disease, consistent with the evidence that NELL1-associated MN is the second most common form of MN, accounting for approximately 10% of cases after the exclusion of autoimmune diseases. Interestingly, results for recent studies identified NELL1 in many secondary forms of MN (malignancies, drugs, sarcoidosis, hepatitis): these pieces of evidence clearly show how NELL1-associated MN overcomes the historical classification in primary and secondary MN [28]. Taken together, the data of the six patients previously described—although relating to a small cohort—seem to show a certain correlation between clinical-histological characteristics and the antigen identified by LMD/MS. Based on this observation and the data already present in the literature, it appears reasonable—pending a wide diffusion of LMD/MS methods—to support a targeted approach already proposed [14, 15] in which the clinical-histological characteristics guide the search for the antigens involved. IHC/IF methods have advantages in that they are cost-effective, and renal pathology laboratories can modify and use existing IHC/IF methodology to stain for individual antigens. IHC/IF tests exist for PLA2R and THSD7A in many laboratories, and more recently, NELL1, EXT1/EXT2, SEMA3B and PCDH7A have been added to the list of MN antigens that can be identified by IHC. Following a targeted approach, IHC/IF test for PLA2R should be routinely performed to identify PLA2R-associated MN and subsequently, where available, other antigens should be tested. However, IHC/IF for many of the other antigens is not practical for several reasons and, at this time is not available for uncommon antigens representing 30%–40% of MN cases. To overcome this limitation, a 1-stop laser microdissection and mass spectrometry clinical test to identify both the common and uncommon antigens of MN has been described recently [29].

The most significant novel finding present in the analysis of our cohort is certainly the one deriving from the six patients in whom LMD/MS showed moderate to high spectral counts for proteins that could be considered putative antigens for MN. The term “putative antigens” has been proposed by authors in order to describe proteins identified on LMD/MS in cases of MN negative for several already known antigens. These proteins are unique, are absent in control cases, have moderate to high total spectral counts and likely represent novel target antigens. Caza *et al*. described seven putative antigens in a cohort of 142 MN cases that were negative for PLA2R, THSD7A, EXT1/2 and NELL1 [30], while Sethi and Madden described 13 putative antigens (three of which have already been described by Caza *et al*.) in a cohort of 250 PLA2R-negative MN [31]. Although together, these antigens may represent approximately 10% of MN cases, taken individually, each of them is extremely rare (<2%), making it difficult to identify the clinical and histological characteristics of the forms of MN associated with putative antigens. Among our cases, four patients were positive for already described putative antigens (PGLYRP1, SULF1, SEZ6L2), while two patients showed putative antigens that have not been previously described: HYAL1 and THBS1. HYAL1 is the primary endoglycosidase that degrades hyaluronic acid. The *HYAL1* gene resides in chromosome 3p21.3 and is associated with tumor suppression, suggesting its possible role in regulating cell proliferation [32]. To the best of our knowledge, HYAL1 has not previously been associated with glomerular disease. THBS1 belongs to a family of five secreted glycoproteins encoded by separated genes. It is released from activated platelets and is also synthesized and secreted by many cell types, including kidney mesangial cells and podocytes. *In vivo*, animal and human data support the important role of THBS1 in podocyte injury and the development of proteinuric kidney disease [33]. Interestingly, the prevalence of putative antigens in our cohort was significantly greater than assumed (6/12 PLA2R-negative MN), likely due to the small cohort size, although a role of the characteristic genetic background of the Sardinian population cannot be excluded.

Our study has several limitations. The most important is the small size of our cohort, which did not allow the evaluation of the prevalence of different forms of MN. Secondly, anti-PLA2R research in serum was not performed using an association of IFA and ELISA in those cases where ELISA showed values >2 RU/mL and <20 RU/mL, possibly leading to an overestimation of serum-PLA2R negative patients.

In conclusion, besides its limitations, our study highlights at least two interesting considerations. First, the determination of PLA2R on renal tissue in the diagnosis of PLA2R-associated MN is emphasized: coherently with the literature, 50% of our cases were falsely diagnosed with PLA2R-negative MN based on the serum anti-PLA2R-Abs determination. Therefore, IHC or IF for PLA2R is essential in the setting of the diagnosis of MN. Second, our study showed six patients with MN likely associated with putative antigens, two of them showing new antigens never described before in the literature (HYAL1 and THBS1). This high prevalence of putative antigens in our cohort is not easily explicable and paves the way for evaluating specific factors in the Sardinian population that could explain this evidence. The role of genetic factors in the high prevalence of autoimmune diseases in the Sardinian population has been demonstrated previously [34]. Therefore, a genetic analysis of Sardinian MN patients may identify specific genetic traits contributing to the high prevalence of rare putative antigens.

## Supplementary Material

sfaf115_Supplemental_File

## Data Availability

The data underlying this article are available in the article and in its online supplementary material.
