# Supplementary material for: The importance of identifying new and putative target antigens associated with membranous nephropathy: evidence from a Sardinian cohort
Source: Clin Kidney J. 2025 Apr 18;18(7):sfaf115. doi: 10.1093/ckj/sfaf115 (PMC12214870; doi:10.1093/ckj/sfaf115)
Supplement: sfaf115_Supplemental_File [file sfaf115_supplemental_file.docx]

Table S1. Characteristics of PLA2R-negative MN cohort

| **Pt** | **Sex** | **Age at onset** | **sCr onset** | **eGFR** | **Proteinuria24h onset** | **Albumin**  **onset** | **IF on KB (+)** | **Dominant IgG4** | **Mesangial proliferation/endocapillary proliferation** | **Autoimmune disease** | **Autoantibodies** | **History of cancer** | **Result of screening for malignancies** | **Other disease** | **HbsAg/HCV status** | **Treatment** | **Response to treatment** | **Target antigen identified on LM/MS** |
| --- | --- | --- | --- | --- | --- | --- | --- | --- | --- | --- | --- | --- | --- | --- | --- | --- | --- | --- |
| 1 | F | 45 | 0,47 | 119 | 6,7 | 1,9 | IgG  IgM  IgA  C3  C1q | NO | YES/NO | Rheumatoid arthritis, autoimmune thyroiditis. | ANA 1:640, ENA-SSA positive | None | Negative | None | Neg/Neg | Steroids + MMF | Complete remission | PGLYRP1 |
| 2 | F | 29 | 0,66 | 122 | 3,17 | 3,6 | IgG  IgM  IgA  C3  C1q | NO | YES/NO | SLE  Fibromyalgia | ANA 1:640  Anti-dsDNA positive | None | Negative | Macular oedema | Neg/Neg | Steroids + MMF | Complete remission | EXT1/EXT2 |
| 3 | M | 40 | 0,9 | 111 | 5,4 | 3 | IgG  IgA  IgM  C3 | YES | NO/NO | None | ANA 1:160  Anti-dsDNA borderline | None | Negative | Arterial hypertension | Neg/Neg | Rituximab | Partial remission | SEZ6L2 |
| 4 | M | 66 | 1,37 | 57 | 3,4 | 3,2 | IgG  IgM  IgA  C3  C1q | YES | YES/NO | None | ANA 1:320  Low C3  Anti-dsDNA borderline | None | Negative | Arterial hypertension  Hypertensive cardiomyopathy  OSAS | Neg/Neg | Rituximab | Complete remission | NCAM-1 |
| 5 | M | 16 | 0,6 | 145 | 5,5 | 3,1 | IgG  C3 | YES | YES/NO | Autoimmune thrombocytopenia (6 month of age), alopecia, autoimmune hemolytic anemia (treated with steroids, onset with NS) | None | None | Negative | None | Neg/Neg | Steroids + Rituximab | Complete remission | HYAL1 |
| 6 | F | 56 | 0,8 | 86 | 12,5 | 1,7 | IgG  C3 | YES | NO/NO | Basedow disease | None | Renal cell carcinoma 1 and half years before diagnosis of MN (tumorectomy) | Negative (3 TB CT scans) | None | Neg/Neg | Rituximab | Partial remission | THSD7A |
| 7 | M | 80 | 1,88 | 36 | 9,2 | 1,9 | IgG  C3 | YES | YES/YES | None | None | FOCAL ADENOCARCINOMA OF THE BIG BOWEL | Negative | Arterial hypertension. CAD. Prostatic Hypertropia. MGUS | Neg/Neg | No treatment  Steroids + CYC  Rituximab | Complete spontaneous remission  No response  Complete remission | THSD7A |
| 8 | F | 22 | 0,7 | 125 | 8,45 | 2,5 | IgG | NO | NO/NO | None | None | None | Negative | X frail syndrome | Neg/Neg | Steroids | Complete remission | THBS1 |
| 9 | M | 81 | 1,8 | 37 | 8 | 2,6 | IgG  C3 | NO | NO/NO | None | None | None | Negative | Interstitial lung disease (antiPR3+); Bosniak III renal cyst; Prostatic hypertropia; Erosive gastropathy; Colon diverticulosis. | Neg/Neg | None | Complete spontaneous remission | NELL-1 |
| 10 | F | 76 | 9 | 5 | 13 | 1,9 | IgG  C3 | NO | NO/YES | None | None | None | Negative | Atrial fibrillation;  Arterial hypertension;  Dyslipidemia | Neg/Neg | No treatment  Steroids + CYC | Complete spontaneous remission  No response | PGLYRP1 |
| 11 | M | 57 | 0,9 | 100 | 5,7 | 3,2 | IgG | NO | NO/NO | None | None | None | Negative | Dyslipidemia | Neg/Neg | Steroids + CYC | Complete remission | NELL-1 |
| 12 | F | 58 | 0,75 | 92 | 4,9 | 2,2 | IgG | YES | NO/NO | Autoimmune thyroiditis. | None | None | Negative | Arterial hypertension.  Dyslipidemia.  antiHBc positive | Neg/Neg | None | Complete spontaneous remission | SULF1 |

CYC: cyclophosphamide; ANA: anti nucleus antibodies; OSAS: obstructive sleeping apnea syndrome; CAD: coronary artery disease; MGUS: monoclonal gammopathy of uncertain significance; THSD7A: thrombospondin type 1 domain containing 7A; NELL-1: neural epidermal growth factor-like 1; EXT 1/2: exostosin 1/2; PGLYRP1: peptidoglycan recognition protein 1; THBS1: Thrombospondin 1; SEZ6L2: seizure related 6 homolog like; HYAL1: hyaluronidase 1; SULF1: sulfatase 1.
